# Genetic Diversity and Structure of *Terminalia bellerica* (Gaertn. Roxb.) Population in India as Revealed by Genetic Analysis

**DOI:** 10.3390/plants13040470

**Published:** 2024-02-06

**Authors:** Subramani Umesh Kanna, Kalappan Thangamuthu Parthiban, Kandasamy Senthilraja, Subramanian Venkatesan, Dhandayuthapani Udhaya Nandhini, Shanmugam Mohan Kumar, Manickam Dhasarathan, Palaniyappan Kumaresan, Makkena Jaswanth Sai, Muthurajan Raveendran, Vellingiri Geethalakshmi

**Affiliations:** 1 Directorate of Planning and Monitoring, Tamil Nadu Agricultural University, Coimbatore 641 003, Tamil Nadu, India; umeshkanna.s@tnau.ac.in; 2 Forest College and Research Institute, Tamil Nadu Agricultural University, Mettupalayam 641 301, Tamil Nadu, India; ktparthi2001@gmail.com (K.T.P.); jaswanth.fcri@gmail.com (M.J.S.); 3 Directorate of Research, Tamil Nadu Agricultural University, Coimbatore 641 003, Tamil Nadu, India; senthilrajaens@gmail.com (K.S.); svengat95@gmail.com (S.V.); raveendrantnau@gmail.com (M.R.); 4 Centre of Excellence in Sustaining Soil Health, Anbil Dharmalingam Agricultural College and Research Institute, Trichy 620 027, Tamil Nadu, India; 5 Agro-Climatic Research Centre, Tamil Nadu Agricultural University, Coimbatore 641 003, Tamil Nadu, India; psmohan13@gmail.com (S.M.K.); plantdr.dhasarathan@gmail.com (M.D.); 6 Centre for Water and Geospatial Studies, Tamil Nadu Agricultural University, Coimbatore 641 003, Tamil Nadu, India; acfkumagri@gmail.com

**Keywords:** *Terminalia bellerica*, genetic diversity, heritability, clustering, progeny trial

## Abstract

In this study, an extensive exploration survey of wild progeny was conducted which yielded 18 candidate plus trees (CPTs) of *Terminalia bellerica*. Seeds of these CPTs were collected from diverse locations between 10°54′ and 28°07′ E longitude, and 76°27′ and 95°32′ N latitude, covering 18 different locations across 5 states of the Indian subcontinent. The objective of the progeny trial was to assess genetic associations and variability in growth and physio-chemical characteristics. Significant variations (*p* < 0.05) were observed among the growth traits, encompassing plant height, basal diameter, girth at breast height and volume, as well as physio-chemical characteristics such as leaf length, width, area and chlorophyll content, carotenoids, and protein in the progeny trial. Broad-sense heritability (h^2^_b_) estimates were consistently high, exceeding 80% for all growth and physiological related traits under investigation except for plant height, leaf length, and girth at breast height. A correlation study revealed that selecting based on plant height, leaf area, and girth at breast height effectively enhances *T. bellerica* volume. A moderate genetic advance in percent of the mean (GAM) was observed for most traits, except leaf length, leaf width, girth at breast height, and plant height. Across all 13 traits, phenotypic coefficient of variation (PCV) surpassed genotypic coefficient of variation (GCV). Utilizing principal component analysis (PCA) and dendrogram construction categorized the genotypes into seven distinct groups. In conclusion, the study has demonstrated that targeting girth at breast height and plant height would be a highly effective strategy for the establishment of elite seedling nurseries and clonal seed nurseries for varietal and hybridization programs in the future.

## 1. Introduction

*T. bellerica* (Gaertn.) Roxb. is an impressive deciduous tree known for its rapid growth and substantial size. With its expansive and spherical crown, it can reach remarkable heights of up to 50 m in its native environment, although it generally grows smaller when nurtured. This tree often showcases prominent buttresses and keeps its branches absent for the initial 20 m of its trunk. The bark of *Terminalia* exhibits a distinctive ashy grey color, highlighted by delicate longitudinal cracks. Additionally, the inner bark showcases a subtle yellowish tint. *Terminalia* can be commonly found in native woodlands across several regions of the Indian subcontinent, including West Bengal, Madhya Pradesh, Uttar Pradesh, Maharashtra, Assam, Tamil Nadu, Rajasthan, Karnataka, Kerala, and Punjab [1].

The wood derived from *T. bellerica* is known for its exceptional hardness and can be utilized in various applications. These include minor construction, the creation of grain measurement tools, boat side planks, fodder production, providing food for Tasar silkworms, soap manufacturing, as well as the extraction of gum with demulcent and purgative properties. Additionally, this wood is also used in the production of ayurvedic medicines such as Triphala. Hence, *T. bellerica* has been selected for the ongoing study. Candidate plus trees (CPTs) have been carefully chosen based on their exceptional morphometric characteristics from diverse geographical regions. The objective is to identify offspring with enhanced productivity through systematic tree improvement initiatives.

The establishment and productivity of forest tree plantations heavily depend on the selection of species and seed sources within those species [2]. Thus, it is crucial to grasp the variation within these seed sources to ensure effective tree improvement programs [3]. In order to obtain superior genetic material, tree breeders must evaluate the traits that require enhancement while taking into account their variability in terms of both morphological and biochemical characteristics [4]. Conducting variability studies is an essential requirement for any tree improvement program [5], however, in the case of *T. bellerica*, such studies are still in the early stages of development.

Besides variability, the role of heritability in estimating potential gains from selection programs is of the utmost importance [6]. Understanding the heritability of selected traits is essential [7], hence it is valuable to assess the genetic analysis to determine the heritable components. At present, there is a lack of information about *T. bellerica* in this field.

Evaluating the extent and type of variation in the initial population is essential for enhancing both qualitative and quantitative progress. Historically, assessing genetic diversity in trees involved conducting provenance/progeny tests and utilizing the Mahalanobis D2 statistic [8]. By clustering genotypes, distantly related clusters can be identified for hybridization, leading to improved segregation to facilitate selection of superior groups or individuals. The individuals or groups that demonstrate enhanced energy and enthusiasm can be effectively utilized in planting programs to enhance productivity [9,10,11,12,13].

Previously, the evaluation of genetic diversity relied on investigations that focused on comparative anatomy, morphology, physiology, and biochemistry [3]. However, the introduction of molecular marker techniques has revolutionized our comprehension of tropical tree population genetics. These techniques enable the analysis of protein or DNA polymorphism and have been instrumental in advancing our understanding in this field [4,14,15,16]. The utilization of DNA marker studies in tropical trees has proven to be effective in various applications. These include understanding variations in origin, determining genotypic identity, characterizing germplasm at a molecular level [17], identifying quantitative trait loci [18,19], studying molecular systematics [20,21], and evaluating genetic diversity [12,13,22]. However, there is a noticeable lack of research on these aspects specifically related to *T. bellerica*. In light of the aforementioned information, this document presents a research study that seeks to explore the potential of *T. bellerica* as a feasible alternative to secondary timber genetic resources. The main aim is to tackle the growing demand for raw wood materials within industries that heavily depend on forest resources.

## 2. Results

### 2.1. Growth Traits

The analysis of variances indicated significant variation among all the 18 accessions studied in the measured growth traits at a significance level of *p* < 0.05 (Figure S1). Significant variation in plant height was observed during the growth of progeny from 18 accessions in the *T. bellerica* (Table 1). Compared to other accessions, FCRITB17 (7.19 m) and FCRITB16 (7.17 m) exhibited significantly higher values for plant height. Conversely, a group of seven accessions exhibited lower heights, measuring less than 6.50 m which included FCRITB18 (6.41 m), FCRITB06 (6.39 m), FCRITB04 (6.29 m), and FCRITB112 (6.23 m).

Significant variation was noted among the accessions in terms of the basal diameter, with an observed mean of 43.9 cm occurring. The accession with the highest basal diameter (50.9 cm) was FCRITB03, followed by FCRITB06 (49.5 cm). The FCRITB14 with a basal diameter of 39.2 cm was apparently distinct, and its measurement was significantly lower than all other accessions.

The range of volumes observed in this study varied from 0.1653 m^3^ to 0.2251 m^3^. Among the nine progenies analyzed, namely FCRITB07 (0.2088 m^3^), FCITB 10 (0.1791 m^3^), FCRITB14 (0.1765 m^3^), FCRITB16 (0.2251 m^3^), FCRITB17 (0.1829 m^3^), and FCRITB18 (0.2013 m^3^) exhibited higher volumes compared to the average value obtained from the overall sample. It is worth mentioning that among the identified progeny, FCRITB16 exhibited the highest recorded volume of 0.2251 m^3^, whereas progeny FCRITB12 achieved the lowest volume of 0.1585 m^3^. The length of the leaves was observed to range from 25.83 cm to 28.30 cm, with an overall average of 26.75 cm. Among the 18 progenies, progeny FCRITB15 and FCRITB17 recorded the maximum leaf length at 28.30 cm, while progeny FCRITB11 exhibited the minimum leaf length at 25.83 cm. The leaf area value exhibited variation ranging from 236.63 cm^2^ to 185.99 cm^2^, with an overall mean value of 208.46 cm^2^. Out of the 18 progenies of *T. bellerica*, 7 progenies—FCRITB04, FCRITB07, FCRITB13, FCRITB15, FCRI TB 16, FCRITB17, and FCRITB18—displayed higher leaf areas compared to the general mean. The progeny FCRITB15 recorded the maximum leaf area of (236.63 cm^2^), while the progeny FCRITB06 exhibited the smallest leaf area of (185.99 cm^2^) (Table 1).

### 2.2. Biochemical Traits

The ANOVA revealed prominent variation (*p* < 0.05) among the 18 studied accessions across all the biochemical traits (Figure S2). Among the 18 progeny, FCRITB05 exhibited higher chlorophyll ‘a’ content with a measurement of 1.127 mg/g, while progeny FCRITB18 had the lowest chlorophyll ‘a’ content at 0.489 mg/g. The overall average for chlorophyll ‘a’ content was recorded as 0.743 mg/g. In terms of chlorophyll ‘b’ content, the general mean was determined to be 0.471 mg/g, ranging from 0.213 mg/g to 0.979 mg/g. Seven specific progenies—FCRITB05 (0.879 mg/g), FCRITB06 (0.613 mg/g), FCRITB07 (0.701 mg/g), FCRITB09 (0.594 mg/g), FCRITB10 (0.534 mg/g), FCR IT B11 (0.497 mg/g), and FCRITB13 (0.293 mg/g)—exhibited higher levels of chlorophyll ‘b’ content compared to the overall average value. The total chlorophyll content varied between 0.501 mg/g and 1.943 mg/g. Among the seven progenies—FCRITB05 (1.943 mg/g), FCRITB06 (0.891 mg/g), FCRITB07 (1.012 mg/g), FCRITB09 (0.904 mg/g), FCRITB10 (0.897 mg/g), FCRITB11 (0.919 mg/g), and FCRITB13 (1.009 mg/g)—the maximum total chlorophyll content was observed when compared to the overall average value. Carotenoid content varied from 0.886 mg/g to 0.372 mg/g, with an average value of 0.581 mg/g. Specifically, the progenies FCRITB02 (0.645 mg/g), FCRITB06 (0.663 mg/g), FCRITB08 (0.604 mg/g), FCRITB10 (0.831 mg/g), FCRITB14 (0.618 mg/g), FCRITB15 (0.818 mg/g), and FCRITB17 (0.886 mg/g) exhibited higher carotenoid content compared to the overall average value. Out of the 18 progenies of *T. bellerica*, 10 specific progenies, including FCRITB03, FCRITB04, FCRITB06, FCRITB10, FCRITB11, FCRITB12, FCRITB13, FCRITB14, and FCRITB17, have demonstrated a higher crude protein content in comparison to the overall average (Table 2).

### 2.3. Heritability

In the conducted study, it was observed that all 13 traits demonstrated a significant level of heritability, ranging from 68.11% to 99.94%, as indicated in Table 3. Among the studied traits, crude protein exhibited the highest level of heritability at 99.94%. This was closely followed by chlorophyll a, b, ratio to chlorophyll a, b (99. 76%, 99. 74%, and 99.31% respectively), as well as carotenoid (99.36%).

### 2.4. Genotypic and Phenotypic Variation

The presence of high variability in chlorophyll a, chlorophyll b, chl a/chl b, total chlorophyll, carotenoid, and crude protein is indicated by the highest values of GCV and PCV. The magnitude of phenotypic coefficient of variation (PCV) was greater than the respective genotypic coefficient of variation (GCV) for all the studied traits, albeit with only a slight difference.

In Table 3, the analysis of the genotypic coefficient of variance (GCV) and phenotypic coefficient of variance (PCV) for multiple traits is presented. The findings reveal that, in the majority of cases, the phenotypic coefficient variances (PCVs) are slightly higher than the genotypic coefficient variances (GCVs). This indicates that the traits being studied are relatively less affected by environmental factors. Notably, the highest values for both GCV and PCV were observed in chlorophyll b (47.80% and 47.74%), crude protein (41.49% and 41.48%), total chlorophyll (37.95% and 37.88%), Chl a/Chl b (35. 65% and 35.60%), chlorophyll a (28.55% and 28.45%), and carotenoid (25.36% and 25.28%). On the other hand, the traits of plant height, basal diameter, girth at breast height, volume, leaf length, leaf width, and leaf area showed limited variability as indicated by their low GCV and PCV values.

### 2.5. Genetic Advance

The findings of this investigation revealed all three kinds of genetic advances (low, moderate, and high). Some traits, including leaf length, leaf width, girth at breast height, and plant height displayed genetic advances of less than 10% in Table 3. In contrast, basal diameter, leaf area, and volume exhibited moderate genetic advances ranging from 13.35% to 17.54%. Particularly, biochemical traits demonstrated a high genetic advance, exceeding 20% with a maximum of 98.24% by chlorophyll b. This study uncovers biochemical traits with both high heritability and genetic advance as a percentage of the mean (>50).

### 2.6. Correlation among the Traits

Correlation analysis revealed several significant relationships between mean progenies traits (Table 4). Plant height of provinces was significantly correlated (r = 0.545 *) with carotenoid (Figure S2) and positively correlated with basal diameter (r = 0.194), girth at breast height (r = 0.158), leaf area (r = 0.248), volume (r = 0.413), leaf length (r = 0.205), leaf width (r = 0.194), and crude protein (r = 0.025). Progenies heights were negatively correlated with chlorophyll content.

### 2.7. Principle Component Analysis

**Figure 1:**
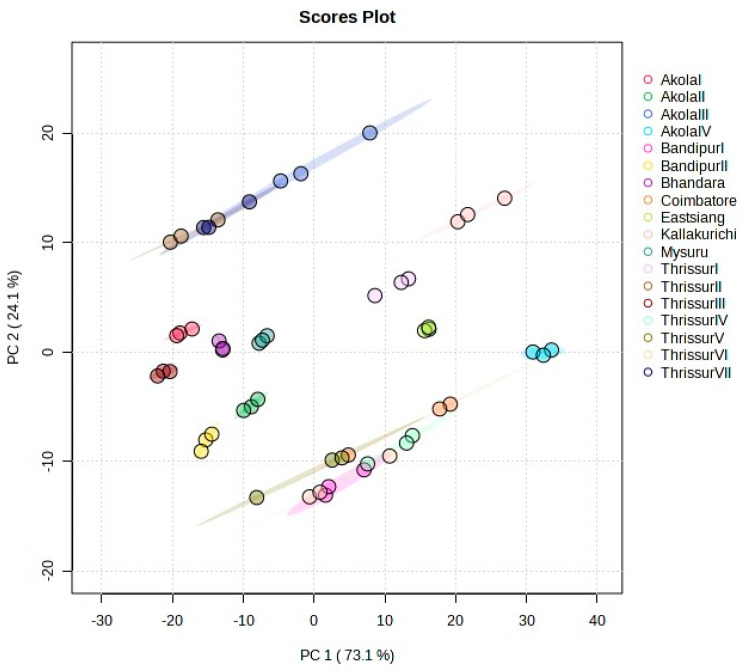
PCA showed the variation in the 18 provinces.

Principle Component Analysis (PCA) was executed to facilitate the visualization of the entire dataset through a condensed dimension plot. The application of PCA was for determining genetic relationships among progenies and exploring correlations among growth, physiological, and biochemical traits. In this study, the performed PCA revealed that over 99% of the observed variances could be accounted for by the initial three principal components (Figure S3). Specifically, PC1, PC2, and PC3 contributed 73.1%, 24.1% and 2.6% to the total variability, respectively (Figure 1. PC1 predominantly represents leaf area and crude protein, PC2 explains the same, and PC3 primarily contributes to basal diameter.

Component scores for the 18 studied progenies are shown in Table S1. Positive values for PC1 indicate progenies with plant height, girth at breast height, leaf area, volume, leaf length, leaf width, and carotenoids in general. FCRITB10, FCRITB16, and FCRITB17 belong to this group. The lowest values for PC1 indicate basal diameter such as FCRITB12 and FCRITB14. The highest values for PC2 indicate all the parameters except for basal diameter and volume. The scatter biplot in Figure S4 shows the relationship between studied genotypes and depicts a clear pattern of the grouping of provincesprovinces. All the provincesprovinces were scattered widely in different quarters.

### 2.8. Heatmap Clustering

**Figure 2:**
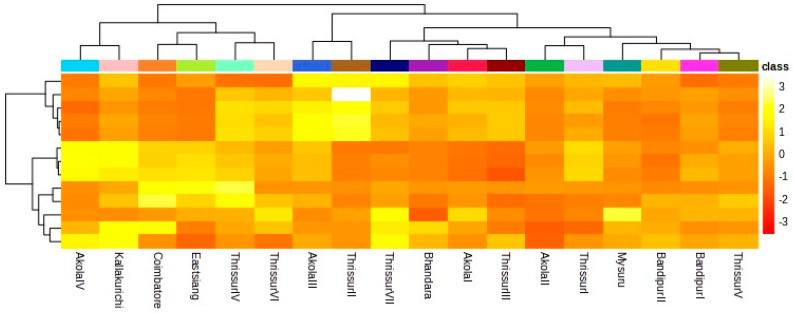
Heatmap dendrogram showed the variations among the 18 progenies.

Figure 2 illustrates K-Means hierarchical clustering for growth characteristics, physiological traits, and biochemical traits in *T. bellerica* accessions. A total of 18 *Terminalia* progenies were categorized into 7 clusters using K-Means clustering, with cluster V and VII holding the highest number of accessions (4) and others sharing 2 progenies under each.

Interestingly, cluster VII consisting of FCRITB01, FCRITB02, FCRITB03, and FCRITB08 exhibited similar mean values for the growth and biophysiological characters. FCRITB15nd FCRITB17 were placed under cluster I and showed similar values for the traits. Despite having the highest number of clustering, progenies did not exhibit high mean values for any of the measured traits amidst the different climatic provincesprovinces.

## 3. Discussion

After a thorough analysis of 18 progenies of *T. bellerica*, it was observed that progenies FCRITB02 and FCRITB13 showed initial superiority based on their biometric attributes. However, progenies FCRITB05, FCRITB07, and FCRITB09 demonstrated significant superiority in more than three biometric traits including plant height, basal diameter, leaf length, leaf width, and leaf area. This highlights the potential of these specific progenies for further study or utilization in future breeding programs.

The progenies, specifically FCRITB03, FCRITB10, FCRITB16, and FCRITB17 displayed significantly higher measurements in various biometric characteristics including height, basal diameter, leaf length, leaf width, and leaf area. The outcomes are consistent with *Neolamarckia cadamba*, *Casuarina* clones, *Ailanthus excelsa*, *Santalum album*, *Dalbergia sissoo*, *Pongamia pinnata*, *Acacia* species, Salix species, *Aquilaria malaccensis*, *Melia azedarach*, *Leucaena leucocephala*, and *Toona ciliata* [7,9,11,23,24,25,26,27,28,29,30,31,32]. Furthermore, the comparable height observed in FCRITB16 and FCRITB17 can be attributed to the similarity in weather parameters, with both provincesprovinces experiencing an average temperature of 17.3 °C and annual rainfall of 2036 mm.

Leaf area is the most essential characteristic when it comes to biomass production. The progenies displayed a significant amount of variation in terms of leaf traits, indicating that these traits can be effectively utilized for selection purposes. The conducted study revealed notable variations in the investigated leaf-related characteristics, including leaf length, leaf breadth, and leaf area. It was observed that among the 18 progenies examined, FCRITB17 demonstrated superiority in all of the analyzed leaf parameters. This could be attributed to its remarkable growth and volume, potentially explaining its exceptional performance. The current investigation is supported by prior evidence showing variation in leaf features and their correlation to production in various plant species such as *Toona ciliata* [33], *N. cadamba* [34,35], *Ficus carica* [36], *Acacia* species [7], *Pongamia pinnata* [37], *Aquilaria malaccensis* [29], Poplar [38], *Dalbergia sissoo* [27], and *Acacia catechu* [39].

Following the analysis of biochemical data from a study on 18 offspring of *T. bellerica*, it was determined that one particular offspring—FCRITB10—consistently displayed significantly elevated levels for all six studied biochemical parameters. Additionally, two other offspring, namely FCRITB12 and FCRITB13, demonstrated superiority in five parameters: chlorophyll ‘a’, chlorophyll ‘b’, chlorophyll a/b ratio, total chlorophyll, and carotenoid levels.

In the present study, the biochemical characteristics of 18 progenies of *T. bellerica* were boserved. Out of the six examined biochemical properties, it is noteworthy that only one progeny, namely FCRITB05, consistently demonstrated superior performance compared to the other progenies. This superiority was observed to be significantly remarkable. Three progenies—FCRITB07, FCRITB10, and FCRITB13—have exhibited their superiority in five biochemical characteristics. These characteristics include chlorophyll ‘a’, chlorophyll ‘b’, chlorophyll a/b ratio, total chlorophyll, and carotenoids. Previous studies have been conducted on various plant species such as *L. leucocephala*, *N. cadamba*, *Ailanthus excelsa*, *Albizia lebbeck*, *Acacia catechu*, *Bassia latifolia*, *Mangifera indica*, and *Ulmis pumila* [40,41,42,43,44,45]. These investigations have shown that these plants exhibit similar variations in terms of their biochemical attributes. Therefore, the findings from previous studies provide support for the conclusions made in the current investigation. Due to its superior performance in a wide range of biometric and biochemical characteristics, FCRITB05 outperformed other progenies of *T. bellerica*. As a result, it is currently being evaluated for prompt integration into future breeding programs.

Heritability serves as a reliable indicator of the transmission of traits from parents to their progeny, categorized as low (below 30%), medium (30–60%), and high (above 60%). The concept of heritability plays a pivotal role in the field of plant breeding, assisting breeders in the selection of genotypes from a wide range of genetic populations. High heritability values are particularly valuable as they enable the effective selection of specific traits. In the conducted study, it was observed that all 13 traits demonstrated a significant level of heritability, ranging from 68.11% to 99.94% as indicated in Table 5. Among the studied traits, crude protein exhibited the highest level of heritability at 99.94%.

The presence of high variability in chlorophyll a, chlorophyll b, chl a/chl b, total chlorophyll, carotenoid, and crude protein is indicated by the highest values of GCV and PCV. The magnitude of phenotypic coefficient of variation (PCV) was greater than the respective genotypic coefficient of variation (GCV) for all the studied traits, albeit with only a slight difference. Similar findings were reported by Rao et al. [46]. In the current study, there was minimal disparity between genotypic and phenotypic coefficients of variation for all the studied traits except plant height. This implies that these traits are less susceptible to environmental influences. Comparable results were reported in *Populus deltoids* [47] and in willow trees [48]. The marginal difference between PCV and GCV of almost all the characters studied in all the traits suggested that there was high heritability of variation among the characters.

Heritability and genetic advancement are pivotal metrics in unraveling the genetic intricacies of various agricultural traits. This study delves into the interplay between heritability and genetic advance as a percentage of mean, shedding light on the potential for effective selection strategies. The analysis reveals that traits exhibiting both high heritability and a high genetic advance as a percentage of the mean primarily operate under the influence of additive gene action. These findings signify the suitability of direct selection to enhance the performance of these traits, promising progress through selective breeding.

Conversely, traits characterized by moderate heritability and low genetic advance as a percentage of the mean are predominantly influenced by non-additive gene action. For such traits, direct selection may pose challenges, as a substantial portion of the variation is attributed to environmental factors. These environmental effects may arise from soil fertility disparities and other unpredictable variables, as suggested by Reddy et al. [49]. Researchers have proposed that traits governed by non-additive gene action may benefit more from management practices than direct selection for trait improvement. This perspective aligns with the recommendations to emphasizing the importance of tailored management approaches [50,51].

The findings of this investigation revealed all three kinds of genetic advances (low, moderate, and high) and uncovers biochemical traits with both high heritability and genetic advance as a percentage of the mean (>50). These high values indicate the prevalence of additive gene action for these specific traits, signifying the potential for effective trait enhancement through selective breeding.

Conversely, the study identifies traits (leaf length, leaf width, girth at breast height, and plant height) characterized by high heritability but low genetic advance as a percentage of the mean. Additionally, traits such as basal diameter, leaf area, and volume exhibit high heritability with moderate genetic advance as a percentage of the mean.

Hierarchical clustering, based on Ward’s minimum variance cluster analysis, revealed phylogeographic patterns of genetic diversity. Length of the horizontal branches between clusters indicates that there is a high degree of dissimilarity between clusters. K-means clustering analysis demonstrated that trees from different geographic regions were grouped together in clusters. Interestingly, trees from the same geographical area were placed in different clusters, suggesting that geographical diversity did not necessarily correlate with genetic diversity and implying that it may have undergone divergent changes in various traits due to different selection pressures. This type of genetic diversity may arise from variations in adoption methods, selection criteria, natural selection pressures, and environmental factors [52]. This suggests that genetic drift has played a more significant role in generating diversity compared to geographic diversity [53]. The absence of any relationships between genetic diversity and geographical distribution in the current study is consistent with the findings of [54,55].

Furthermore, this clustering approach identified promising accessions with favorable traits, paving the way for the establishment of elite seedling nurseries and clonal seed nurseries for varietal and hybridization programs in the future.

The growth of a plant, as indicated by volume, basal diameter, and plant height is considered highly significant for improvement in the current study. Similarly, growth traits in black poplar are the most crucial based on principal component analysis [56]. In a study on the morphological characters of *P. deltoides* hybrid clones in a nursery, Ozel et al. [57] applied factor analysis, explaining 71.46% of the total variance with the first five components and captured 90% cumulative variability for the first two principal components to differentiate leaf characters of *Populus nigra* similar to the present study (0.948) [58]. Tunctaner [59] reported five principal components based on the study of fourteen traits in willow clones, a pattern also observed by Singh et al. [53] in Salix clones. The growth characters are attributed to distinct genetic constitution of the clones as highlighted in this study [60]. The promising clones selected for this study must undergo multi location trials to investigate the relationship between genotype and environment at various sites. This will allow for an analysis of the suitability of the clones and allow for the use of the clones for intra- and inter-specific control breeding (hybridization) aimed at producing more productive clones.

## 4. Materials and Methods

### 4.1. Genetic Material

**Figure 3:**
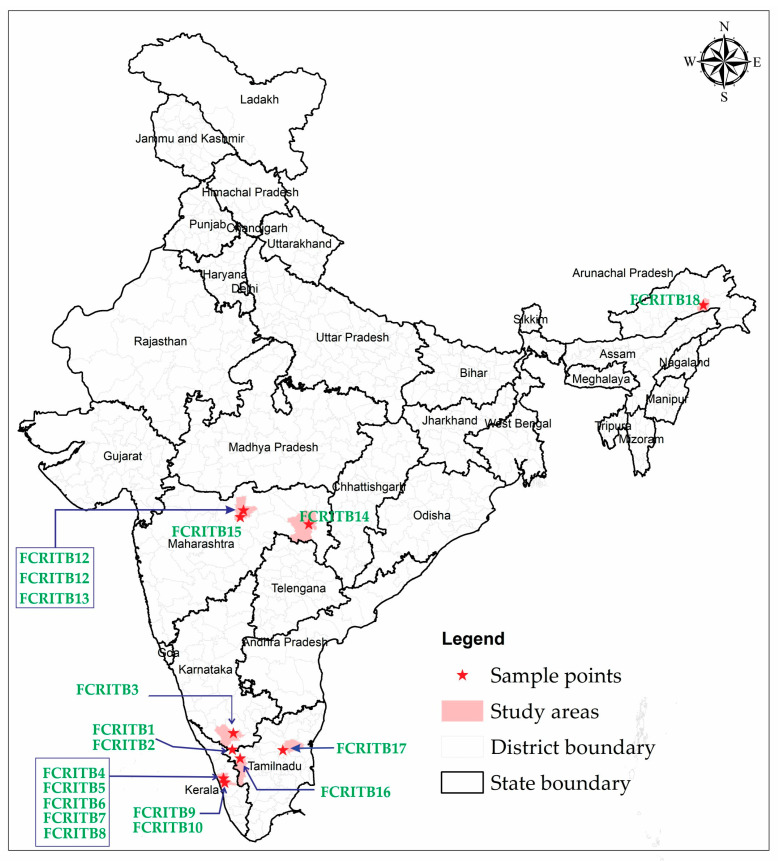
Map showing the progenies collection point.

A comprehensive and extensive survey of wild germplasm was conducted with the aim of identifying promising candidate plus trees (CPTs) of *T. bellerica*. This survey originated from five distinct states of the Indian subcontinent: Tamil Nadu, Maharashtra, Kerala, Karnataka, and Arunachal Pradesh, and examined the ecological impact on genetic diversity, growth, and eco-physiological traits (Figure 3, Table 5). These provinces were selected due to their inherent adaptability to the growing conditions suitable for *T. bellerica.* As all of the selected origins are distributed across the Indian subcontinent, they exhibit both commonalities and variations in their climatic origins. The selection process of CPTs involved utilizing the single-tree selection method, which relied on assessing the phenotypic traits with economic significance *viz*. total height, girth at breast height, bole height, and volume [50] (Figure S5, Table 6). Precautions were taken to ensure that the selected trees were free from pest and disease infestations and excluded isolated or poorly performing trees, commonly referred to as wolf trees. A total of 18 CPTs were collected from diverse locations between 10°54′ and 28°07′ E longitude, and 76°27′ and 95°32′ N latitude, across five states of the Indian subcontinent (Figures S6 and S7). Three kilograms of mature pods were harvested from each CPT by following a random sampling procedure. These pods were collected from all four directions of the crown of each selected tree during the fruiting season between September and November in the year 2019. The gathering of potential CPTs was achieved through collaboration with officials from the respective forest departments while strictly adhering to required permissions and regulations.

### 4.2. Study Site

After the collection process, the progenies were brought to the Forest College and Research Institute (FC&RI), TNAU, located in Mettupalayam, Tamil Nadu, India (geographical coordinate of 11.32° N latitude and 76.93° E longitude, 320 m MSL). Mettupalayam experiences a semi-arid climate characterized by a mean annual rainfall of 945 mm, along with an average of 73.6 rainy days per year. The annual temperature range varies from a minimum of 15.4 °C to a maximum of 34.9 °C. Typically, the lowest temperatures are recorded in January, while the highest temperatures occur in May each year. For the purpose of identifying elite progeny, a trial was initiated in the year 2020 at the FC&RI with three replications.

### 4.3. Progenies Planting

The plus trees’ seeds were planted in raised beds, utilizing a mixture of red soil, sand, and farmyard manure (FYM) in a 2:1:1 ratio. These beds were consistently watered and meticulously tended to for a duration of two months. Following this period, the saplings with a collar region thickness exceeding 3–4 cm were carefully chosen and transplanted into polybags containing a blend of red soil, sand, and FYM in the same 2:1:1 ratio. Approximately one month after transplantation, these young seedlings were finally transferred and planted in the main field. No treatments or fertilizers were applied during the nursery stage. The establishment of the progeny evaluation trial in the field adhered to a randomized block design (RBD), with plants spaced at intervals of 4 × 4 m. Within each replication, four progenies per CPT were included for comprehensive evaluation. During the planting process, each seedling received additional nutrients in the form of 250 g of farmyard manure (FYM), 25 g of vermicompost and 5 g of di-ammonium phosphate (DAP). The subsequent data was acquired from the trees that were planted and observed at different time intervals.

### 4.4. Morphological

Data were meticulously recorded for all 18 progenies within each replication when the plants were 24 months old for the morphological traits. Field measurements were taken for each individual, including tree height (H) and basal diameter (BD). The plant’s height was measured in meters (m) from the base of the stem to the tip using a measuring tape. The basal diameter of the trees at their base (in centimeters) was measured using a digital caliper from the Large SDN series. In cases where a tree had multiple basal stems, the diameters of all individual trunks were measured, and a single equivalent basal diameter (BD) value was calculated following the method outlined by Alvarez et al. [61].

### 4.5. Biochemical Parameters

Chlorophyll was extracted from fresh leaves using 80% acetone and 0.25 g leaf samples. The resulting extract was then measured spectrophotometrically at wavelengths of 475 nm, 645 nm, and 663 nm. The determination of total chlorophyll and carotenoid contents was carried out using established methodologies [62]. The Lowrey’s method [63] was employed to evaluate the protein content of the leaves.

### 4.6. Genetic Estimates

Heritability, genetic advancement as a percentage of the mean, phenotypic, and genotypic coefficients of variation (PCV and GCV), were calculated for volume as well as growth traits, following the methodologies proposed by various researchers [64,65,66].

Broad-sense heritability in all the progenies was estimated by dividing the variance in measurements into two components: between-accessions and within-accessions [67].

### 4.7. Statistical Analysis

The initial dataset was created by calculating the averages for each trait across four CPTS within each replication and between replication in the experiment. These calculated means were then subjected to subsequent statistical and genetic analyses. Correlation between traits to reveal possible associations was calculated with raw data based on single plant estimates, using the Pearson correlation coefficient at *p* ≤ 0.05. PCA was performed with progeny means to determine the relationships among progenies and to obtain an overview of correlation among traits. Various statistical analysis was conducted using the SPSS Windows software package (IBM SPSS version 26).

## 5. Conclusions

The ultimate objective of tree improvement is to enhance the growth and yield traits of tree species. These traits are intricate and are influenced by the interaction of various physiological and morphological characteristics. Therefore, solely relying on the performance of individual tree species for improvement might prove to be less effective. Hence, it can be concluded that for tree improvement of *T. bellerica* through the phenotypic selection process, the number of plus trees selected from a population should be sufficiently large in order to exploit the large intra-population genetic variation. Besides, significant differences were found between the features in the progeny study, which evaluated genetic correlations and variability in growth and physio-chemical parameters. For the majority of variables, estimates of broad-sense heritability were high, suggesting significant genetic control. Plant height, leaf area, and girth at breast height were found to be important characteristics for increasing *T. bellerica* volume through correlation studies. The study demonstrated the effectiveness of targeting girth at breast height and plant height for establishing elite seedling nurseries and clonal seed nurseries for future varietal and hybridization programs.

## Figures and Tables

**Table 1:** Mean performance of selected genotypes for growth and physiological traits in *T. bellerica*.

Accession Name	Plant Height (m)	Basal Diameter (cm)	Girth at Breast Height (m)	Volume (m^3^)	Leaf Length (cm)	Leaf Width (cm)	Leaf Area (cm^2^)
FCRITB01	6.50 ± 0.14 ^efgh^	44.2 ± 0.63 ^d^	0.09 ± 0.001 ^b^	0.692 ± 0.00 ^ef^	26.5 ± 0.10 ^defg^	11.6 ± 0.01 ^de^	208 ± 1.88 ^ef^
FCRITB02	6.67 ± 0.11 ^cdef^	43.6 ± 0.00 ^de^	0.09 ± 0.000 ^b^	0.690 ± 0.00 ^de^	25.9 ± 0.54 ^efg^	11.0 ± 0.17 ^gh^	194 ± 0.58 ^jk^
FCRITB03	6.73 ± 0.16 ^cde^	50.9 ± 0.81 ^a^	0.09 ± 0.000 ^b^	0.631 ± 0.00 ^hij^	26.6 ± 0.26 ^cdef^	11.2 ± 0.21 ^fgh^	201 ± 0.35 ^fgh^
FCRITB04	6.29 ± 0.09 ^gh^	41.6 ± 0.40 ^gh^	0.09 ± 0.001 ^b^	0.625 ± 0.01 ^ij^	27.5 ± 0.30 ^ab^	11.9 ± 0.09 ^abcd^	222 ± 1.51 ^bc^
FCRITB05	6.53 ± 0.03 ^defg^	43.4 ± 0.73 ^def^	0.09 ± 0.001 ^b^	0.627 ± 0.00h ^ij^	25.9 ± 0.38 ^fg^	11.1 ± 0.00 ^fgh^	195 ± 2.13 ^ij^
FCRITB06	6.39 ± 0.09 ^fgh^	41.9 ± 0.00 ^fg^	0.09 ± 0.001 ^b^	0.594 ± 0.00 ^k^	25.6 ± 0.01 ^g^	10.7 ± 0.01 ^i^	186 ± 0.52 ^k^
FCRITB07	6.65 ± 0.07 ^cdef^	44.4 ± 0.85 ^d^	0.10 ± 0.000 ^a^	0.775 ± 0.00 ^b^	27.1 ± 0.39 ^bcd^	11.8 ± 0.03 ^bcd^	218 ± 2.10 ^cd^
FCRITB08	6.53 ± 0.09 ^defg^	44.4 ± 0.06 ^d^	0.09 ± 0.002 ^b^	0.720 ± 0.00 ^c^	26.7 ± 0.01 ^bcde^	11.3 ± 0.10 ^efg^	205 ± 3.99 ^fg^
FCRITB09	6.80 ± 0.09 ^bcd^	47.6 ± 0.18 ^bc^	0.09 ± 0.001 ^b^	0.711 ± 0.02 ^cd^	26.8 ± 0.11 ^cdef^	11.4 ± 0.12 ^ef^	207 ± 3.73 ^ef^
FCRITB10	7.04 ± 0.14 ^ab^	49.5 ± 0.15 ^b^	0.09 ± 0.000 ^b^	0.663 ± 0.01 ^fg^	26.1 ± 0.10 ^fg^	11.3 ± 0.05 ^fgh^	199 ± 2.18 ^ghi^
FCRITB11	6.63 ± 0.15 ^def^	46.7 ± 0.19 ^c^	0.09 ± 0.002 ^b^	0.648 ± 0.01 ^gh^	25.8 ± 0.25 ^fg^	10.9 ± 0.03 ^hi^	192 ± 0.70 ^jk^
FCRITB12	6.23 ± 0.11 ^h^	40.3 ± 0.51 ^h^	0.09 ± 0.001 ^b^	0.613 ± 0.01 ^jk^	26.4 ± 0.11 ^cdefg^	11.2 ± 0.22 ^fgh^	201± 0.60 ^ghi^
FCRITB13	6.55 ± 0.06^defg^	41.7 ± 0.52 ^fg^	0.09 ± 0.000 ^b^	0.691 ± 0.01 ^e^	27.1 ± 0.14 ^bc^	11.6 ± 0.22 ^cde^	213 ± 4.08 ^de^
FCRITB14	6.94 ± 0.03^abc^	39.2 ± 0.25 ^i^	0.09 ± 0.001 ^b^	0.616 ± 0.01 ^ijk^	26.0 ± 0.61 ^efg^	11.1 ± 0.02 ^fgh^	196 ± 0.06 ^hij^
FCRITB15	6.73 ± 0.13 ^cde^	42.1 ± 0.06 ^fg^	0.09 ± 0.001 ^b^	0.639 ± 0.00 ^hi^	28.3 ± 0.34 ^a^	12.3 ± 0.04 ^a^	237 ± 0.75 ^a^
FCRITB16	7.17 ± 0.07 ^a^	42.5 ± 0.45 ^efg^	0.10 ± 0.002 ^a^	0.819 ± 0.00 ^a^	27.7 ± 0.11 ^b^	11.9 ± 0.00 ^abc^	220 ± 4.81 ^bcd^
FCRITB17	7.19 ± 0.01 ^a^	41.9 ± 0.26 ^fg^	0.09 ± 0.000 ^b^	0.708 ± 0.00 ^cde^	28.3 ± 0.20 ^a^	12.2 ± 0.23 ^ab^	234 ± 2.13 ^a^
FCRITB18	6.41 ± 0.02 ^fgh^	43.7 ± 0.48 ^de^	0.10 ± 0.001 ^a^	0.731 ± 0.02 ^c^	27.3 ± 0.14 ^b^	12.2 ± 0.04 ^ab^	225 ± 0.21 ^b^

Note: Data are the mean values of three replicates with ± standard error. Means followed by the same letter within each column are not significantly different at the 0.05 level.

**Table 2:** Mean performance of selected genotypes for biochemical traits in *T. bellerica*.

Accession Name	Chlorophyll a (mg/g)	Chlorophyll b (mg/g)	Chlorophyll a and b	Total Chlorophyll (mg/g)	Carotenoid mg/g	Crude Protein%
FCRITB01	0.628 ±0.01 ^g^	0.435 ±0.00 ^i^	1.063 ±0.00 ^i^	0.754 ±0.00 ^e^	0.561 ±0.00 ^f^	11.59 ±0.19 ^f^
FCRITB02	0.552 ±0.01 ^h^	0.213 ±0.00 ^n^	0.765 ±0.01 ^l^	0.690 ±0.00 ^f^	0.645 ±0.00 ^d^	21.11 ±0.31 ^d^
FCRITB03	0.515 ±0.00 ^i^	0.278 ±0.00 ^l^	0.793 ±0.00 ^k^	0.654 ±0.01 ^g^	0.574 ±0.01 ^f^	27.78 ±0.08 ^f^
FCRITB04	0.771 ±0.02 ^f^	0.398 ±0.00 ^j^	1.169 ±0.01 ^h^	0.798 ±0.01 ^d^	0.513 ±0.00 ^g^	26.26 ±0.00 ^g^
FCRITB05	1.127 ±0.02 ^a^	0.979 ±0.01 ^a^	2.106 ±0.00 ^a^	1.943 ±0.04 ^a^	0.518 ±0.00 ^g^	39.88 ±0.07 ^g^
FCRITB06	0.826 ±0.00 ^e^	0.613 ±0.01 ^d^	1.439 ±0.02 ^e^	0.891 ±0.00 ^c^	0.663 ±0.00 ^d^	29.18 ±0.10 ^d^
FCRITB07	0.926 ±0.01 ^c^	0.701 ±0.01 ^c^	1.627 ±0.03 ^c^	1.012 ±0.00 ^b^	0.513 ±0.01 ^g^	12.15 ±0.14 ^g^
FCRITB08	0.519 ±0.01 ^i^	0.298 ±0.00 ^k^	0.817 ±0.02 ^k^	0.649 ±0.00 ^g^	0.604 ±0.01 ^e^	13.88 ±0.09 ^e^
FCRITB09	0.892 ±0.01 ^d^	0.594 ±0.01 ^e^	1.486 ±0.00 ^d^	0.904 ±0.01 ^c^	0.416 ±0.00 ^h^	11.73 ±0.02 ^h^
FCRITB10	0.836 ±0.00 ^e^	0.534 ±0.01 ^f^	1.370 ±0.00 ^f^	0.897 ±0.01 ^c^	0.831 ±0.01 ^b^	39.39 ±0.13 ^b^
FCRITB11	0.823 ±0.02 ^e^	0.497 ±0.00 ^g^	1.320 ±0.01 ^g^	0.919 ±0.01 ^c^	0.498 ±0.00 ^g^	31.40 ±0.05 ^g^
FCRITB12	0.562 ±0.00 ^h^	0.301 ±0.00 ^k^	0.863 ±0.00 ^j^	0.619 ±0.00 ^h^	0.364 ±0.00 ^i^	22.16 ±0.12 ^i^
FCRITB13	0.997 ±0.01 ^b^	0.923 ±0.00 ^b^	1.920 ±0.01 ^b^	1.009 ±0.00 ^b^	0.574 ±0.00 ^f^	39.88 ±0.14 ^f^
FCRITB14	0.615 ±0.01 ^g^	0.464 ±0.00 ^h^	1.079 ±0.01 ^i^	0.710 ±0.00 ^f^	0.618 ±0.01 ^e^	28.45 ±0.30 ^e^
FCRITB15	0.436 ±0.00 ^j^	0.254 ±0.00 ^m^	0.690 ±0.00 ^m^	0.593 ±0.00 ^h^	0.818 ±0.00 ^c^	13.88 ±0.23 ^c^
FCRITB16	0.513 ±0.00 ^i^	0.294 ±0.00 ^kl^	0.807 ±0.00 ^k^	0.604 ±0.00 ^h^	0.498 ±0.01 ^g^	13.64 ±0.02 ^g^
FCRITB17	0.612 ±0.00 ^g^	0.454 ±0.01 ^hi^	1.066 ±0.01 ^i^	0.784 ±0.01 ^d^	0.886 ±0.01 ^a^	29.09 ± 0.07 ^a^
FCRITB18	0.489 ±0.00 ^i^	0.254 ±0.00 ^m^	0.743 ±0.02 ^l^	0.501 ±0.01 ^i^	0.372 ±0.00 ^i^	21.31 ± 0.06 ^i^

Traits marked with the same superscript letter are not statistically significantly different at a significance level of *p* = 0.05.

**Table 3:** Genetic estimates of selected progeny traits in *T. bellerica*.

	Traits	Phenotypic Coefficient of Variation	Genotypic Coefficient of Variation	Heritability Broad Sense (%)	GA (%) of Mean
Growth traits	Plant height (m)	4.70	3.88	68.11	6.59
	Basal diameter (cm)	6.94	6.71	93.37	13.35
	Girt at breast height (m)	4.69	4.16	78.68	7.61
	Volume (m^3^)	9.01	8.76	94.54	17.54
Physiological traits	Leaf length (cm)	3.47	2.92	71.00	5.07
	Leaf Width (cm)	4.21	3.80	81.65	7.08
	Leaf area (cm^2^)	7.34	7.08	92.98	14.06
Biochemical traits	Chlorophyll a (mg/g)	28.55	28.45	99.31	58.41
	Chlorophyll b (mg/g)	47.80	47.74	99.76	98.24
	Chl a/Chl b	35.65	35.60	99.74	73.25
	Total Chlorophyll (mg/g)	37.95	37.88	99.63	77.89
	Carotenoid (mg/g)	25.36	25.28	99.36	51.91
	Crude Protein (%)	41.49	41.48	99.94	85.42

**Table 4:** Pearson Correlation Coefficient among different characters studied.

	PH		GBH	LA	Volume	Leaf Length	Leaf Width	Chl a	Chl b	Chl a/Chl b	Total Chlorophyll	Carotenoid	Crude Protein
PH	1	0.194	0.158	0.248	0.413	0.205	0.194	−0.136	−0.064	−0.096	−0.087	0.545 *	0.025
BD		1	−0.020	−0.188	0.119	−0.284	−0.231	0.088	−0.044	0.026	0.036	0.007	0.023
GBH			1	0.383	0.747 **	0.317	0.489 *	−0.096	−0.082	−0.088	−0.172	−0.312	−0.321
LA				1	0.416	0.962 ^**^	0.973 **	−0.313	−0.203	−0.257	−0.304	0.220	−0.336
Volume					1	0.366	0.523 *	−0.105	−0.075	−0.086	−0.176	−0.150	−0.504 *
Leaf length						1	0.932 **	−0.386	−0.272	−0.331	−0.358	0.215	−0.302
Leaf Width							1	−0.323	−0.223	−0.273	−0.322	0.072	−0.410
Chl a								1	0.938 **	0.983 **	0.852 **	−0.122	0.514 *
Chl b									1	0.985 **	0.850 **	−0.034	0.529 *
Chl a/Chl b										1	0.866 **	−0.082	0.523 *
Total Chlorophyll											1	−0.042	0.513 *
Carotenoid												1	0.256
Crude Protein													1

**. Correlation is significant at the 0.01 level (two-tailed) *. Correlation is significant at the 0.05 level (two-tailed). Note: PH—Plant height; BD—Basal diameter; GBH—Girth at breast height; LA—Leaf area.

**Table 5:** Provinces selected from the Indian subcontinent and the geographical location details.

S.No	Sources	District	State	Latitude	Longitude	Assigned Name
1.	Bandipur Tiger Reserve and National Park	Bandipur	Karnataka	11.664547	76.626421	FCRITB1
2.	Bandipur Tiger Reserve and National Park	Bandipur	Karnataka	11.664571	76.626418	FCRITB2
3.	Mysuru Zoo	Mysuru	Karnataka	12.30053	76.669647	FCRITB3
4.	Kerala Agricultural University	Thrissur	Kerala	10.3832	76.3296	FCRITB4
5.	Kerala Agricultural University	Thrissur	Kerala	10.3836	76.3299	FCRITB5
6.	Vellanikkara	Thrissur	Kerala	10.548235	76.278912	FCRITB6
7.	Vellanikkara	Thrissur	Kerala	10.548007	76.278745	FCRITB7
8.	Vellanikkara	Thrissur	Kerala	10.54824	76.278874	FCRITB8
9.	Bentham and Hooker Garden	Thrissur	Kerala	10.547665	76.278592	FCRITB9
10.	Bentham and Hooker Garden	Thrissur	Kerala	10.550524	76.280483	FCRITB10
11.	Akola	Akola	Maharashtra	20.703063	77.069286	FCRITB11
12.	Akola	Akola	Maharashtra	20.703088	77.069316	FCRITB12
13.	Akola	Akola	Maharashtra	20.703003	77.069991	FCRITB13
14.	Shioni	Bhandara	Maharashtra	20.191579	79.661286	FCRITB14
15.	Patur	Akola	Maharashtra	20.461537	76.943464	FCRITB15
16.	Jagnari slopes	Coimbatore	Tamil Nadu	11.323315	76.934989	FCRITB16
17.	Kalarayan Hills	Kallakurichi	Tamil Nadu	11.764162	76.415564	FCRITB17
18.	Pasighat	Eastsiang	Arunachal Pradesh	28.075837	95.325901	FCRITB18

**Table 6:** Morphometric attributes of selected Candidate Plus Trees of *T. bellerica*.

Accession Name	GBH (m)	Height (m)	Clear Bole Height (m)	Volume (m^3^)
FCRITB01	3.7	17.0	8.2	731.00
FCRITB02	3.2	17.2	6.1	553.21
FCRITB03	0.90	12.0	5.7	30.53
FCRITB04	1.36	19.0	11.6	110.38
FCRITB05	1.45	18.0	9.8	118.87
FCRITB06	1.39	18.5	11.2	112.27
FCRITB07	1.90	17.3	10.4	196.16
FCRITB08	1.76	21.0	9.7	204.32
FCRITB09	2.4	19.3	10.7	349.17
FCRITB10	1.8	17.6	12.4	179.11
FCRITB11	2.8	9.2	3.6	226.55
FCRITB12	1.70	9.0	4.7	81.69
FCRITB13	1.40	11.0	3.3	67.71
FCRITB14	1.80	10.6	2.8	107.87
FCRITB15	1.40	10.2	4.7	62.79
FCRITB16	2.34	22.0	10.6	378.37
FCRITB17	2.30	23.0	7.9	204.37
FCRITB18	9.7	9.7	4.2	121.87

## Data Availability

The data is available from corresponding author upon reasonable request.

## Supplementary Materials

The following supporting information can be downloaded at: https://www.mdpi.com/article/10.3390/plants13040470/s1, Figure S1: One way ANOVA plot showing the significant differences among the studied physiochemical properties amidst the eighteen progenies; Figure S2: Correlation map showing the relationship between 13 characters in the study; Figure S3: PCA variance explained; Figure S4: Segregation of the 18 Terminalia progenies according to their growth, physiological and biochemical characteristics determined by principal component analysis; Figure S5: Selection of candidate plus; Figure S6: Walter-Leith diagram of the monthly rainfall and daily average temperature of Bandipur (Karnataka), Thrissur (Kerala), Kallakurichi (Tamil Nadu) and Jognari (Tamil Nadu); Figure S7: Walter-Leith diagram of the monthly rainfall and daily average temperature of Maharashtra, Mysuru (Karnataka), Pasighat (Arunachal Pradesh) and Vellanikkara (Kerala); Table S1: Component scores and loadings of *T. bellerica* traits.
