# Supplementary material for: Genetic Diversity and Structure of Terminalia bellerica (Gaertn. Roxb.) Population in India as Revealed by Genetic Analysis
Source: Plants (Basel). 2024 Feb 6;13(4):470. doi: 10.3390/plants13040470 (PMC10892032; doi:10.3390/plants13040470)
Supplement: Supplementary file 1 [file plants-13-00470-s001.zip › plants-2768497-supplementary.pdf]

Supplementary Materials

Table S1. Component scores and loadings of *T. bellerica* traits

	**PC1**	**PC2**	**PC3**	**PC4**	**PC5**
**Variance %**	**73.1**	**24.1**	**2.6**	**0.1**	**0.1**
PH	0.004	0.005	-0.024	0.03	-0.126
**BD**	-0.034	-0.027	-0.998	0.019	0.025
**GBH**	0	0	0	0	0.001
**LA**	0.948	0.311	-0.042	-0.038	-0.034
**Vol**	0.002	-0.002	-0.003	-0.016	0.02
**LL**	0.048	0.016	0.027	0.684	0.721
**LW**	0.027	0.003	0.007	0.022	0.07
**Chl a**	-0.005	0.008	-0.003	-0.248	0.216
**Chl b**	-0.004	0.011	0.004	-0.276	0.252
**Chl a/Chl b**	-0.009	0.019	0	-0.522	0.479
**Total C**	-0.007	0.013	0	-0.342	0.338
**Car**	0.001	0.006	-0.003	0.049	-0.064
**CP**	-0.312	0.95	-0.015	0.021	-0.019

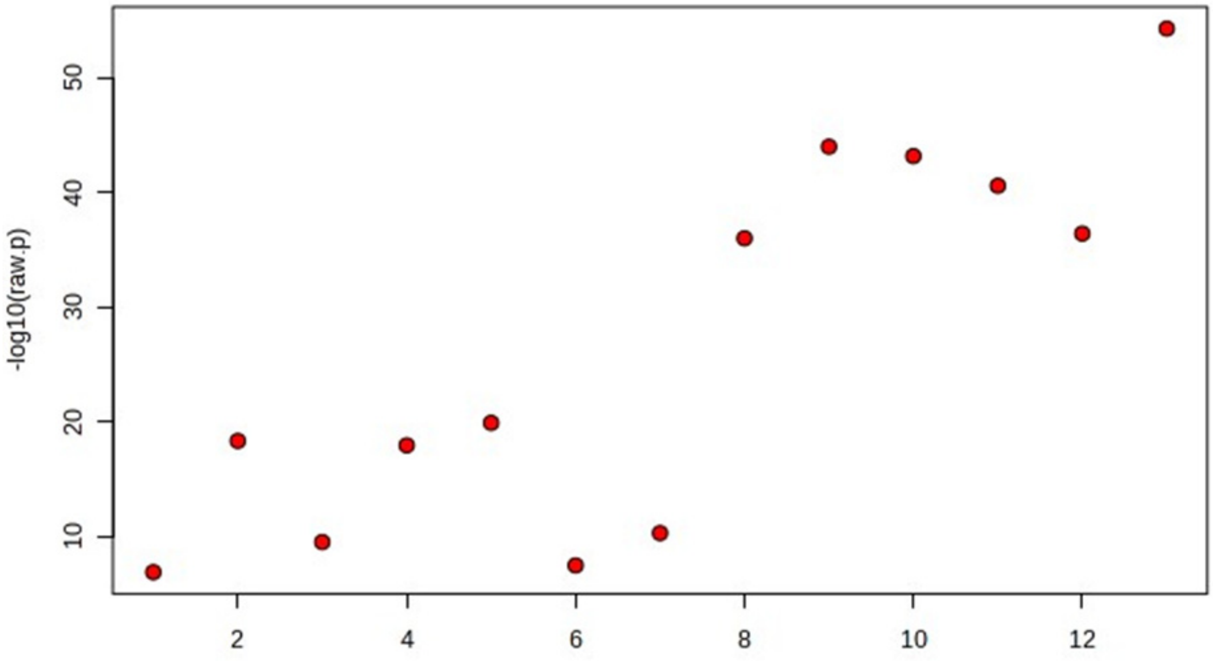

Figure S1. One way ANOVA plot showing the significant differences among the studied physiochemical properties amidst the eighteen progenies.

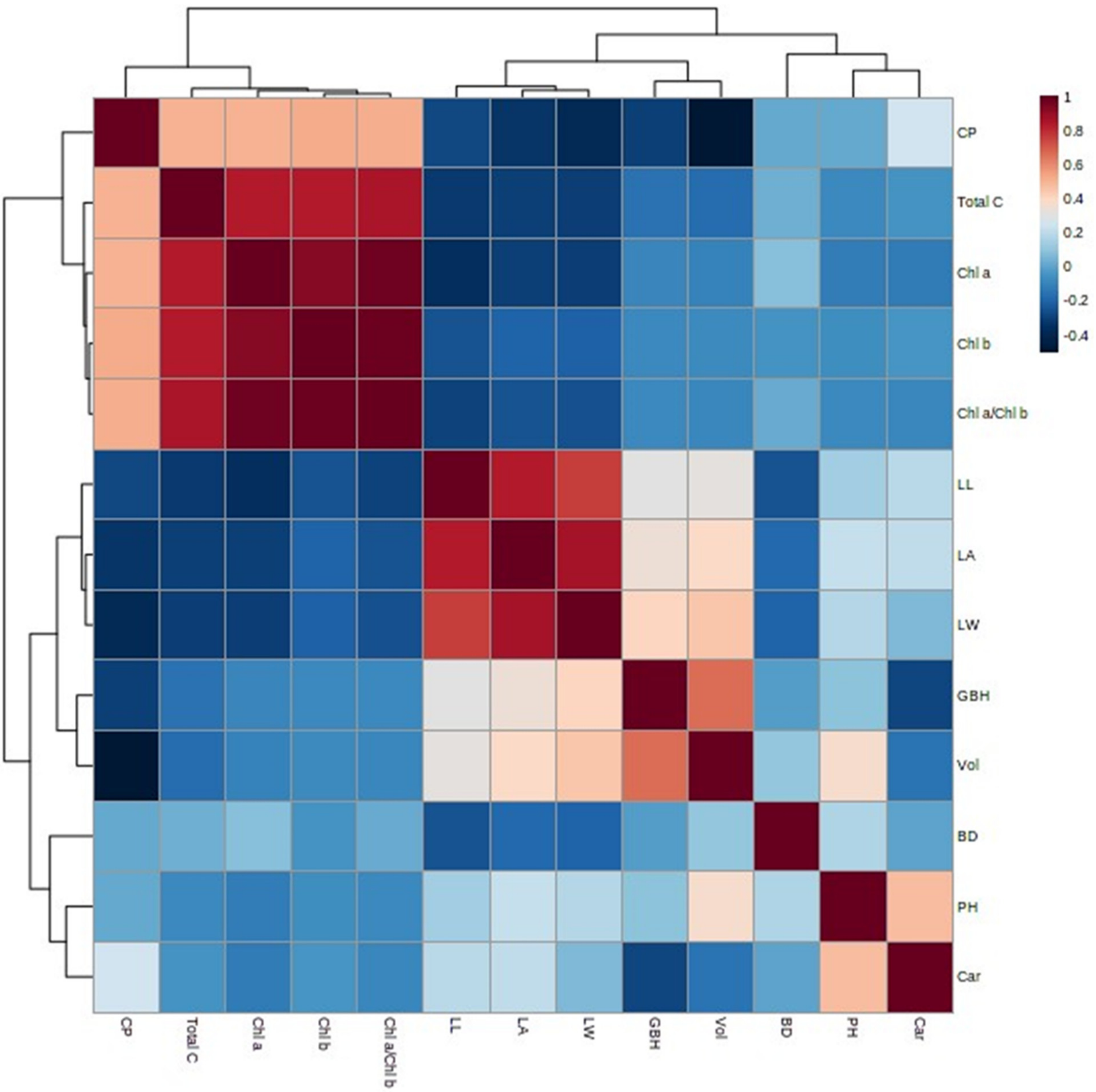

Figure S2. Correlation map showing the relationship between 13 characters in the study

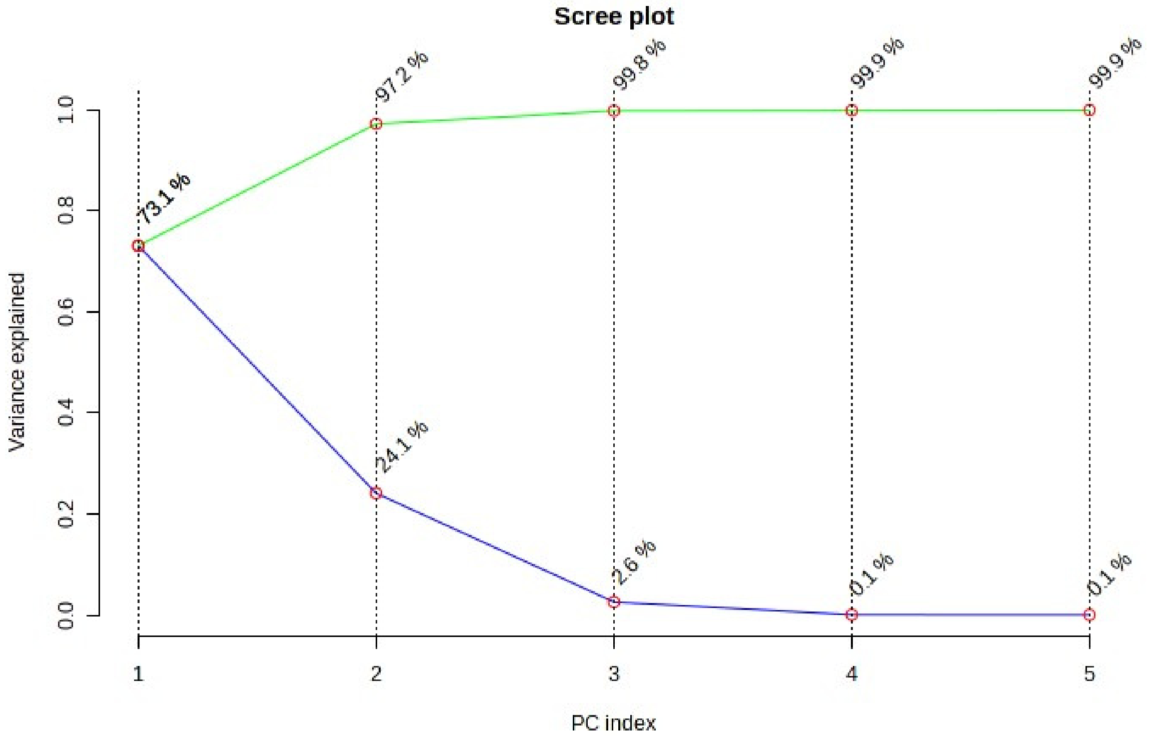

Figure S3. PCA variance explained

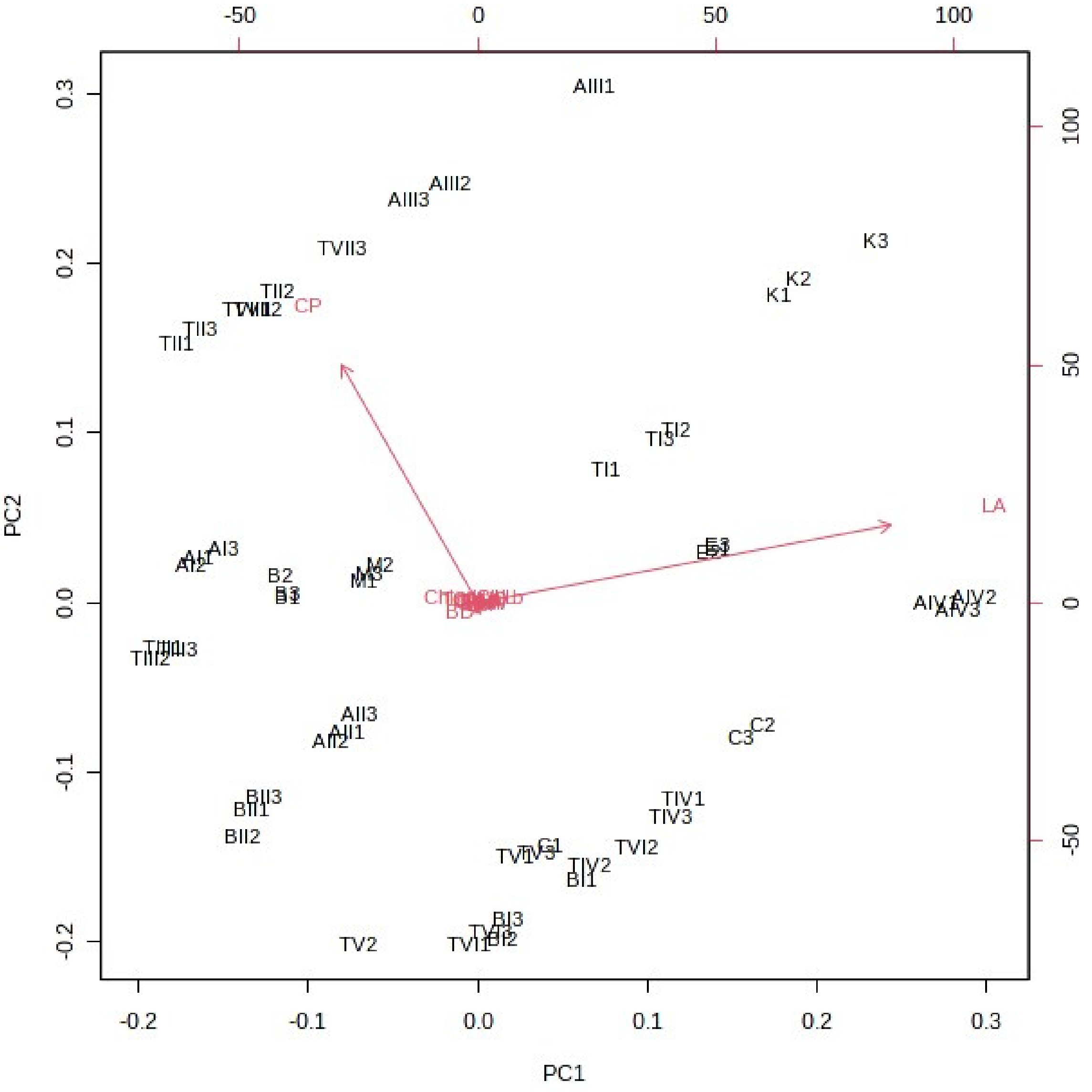

Figure S4. Segregation of the 18 *Terminalia* progenies according to their growth, physiological and biochemical characteristics determined by principal component analysis.

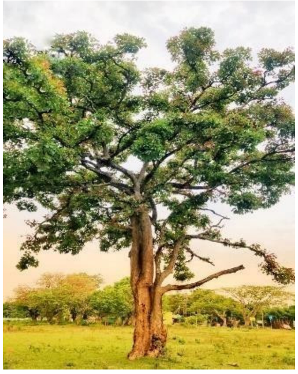
FCRI TB1

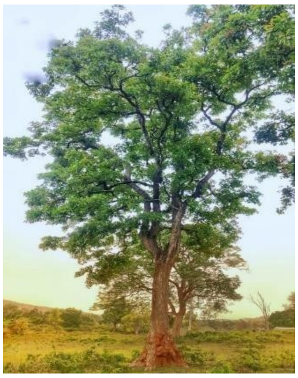
FCRI TB2

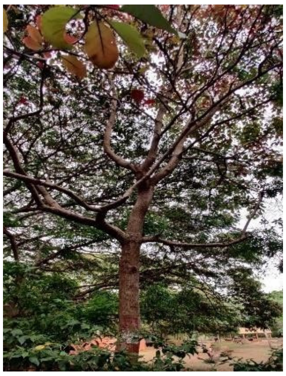
FCRI TB3

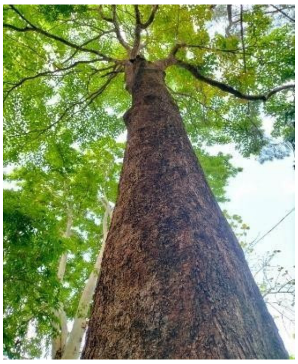
FCRI TB4

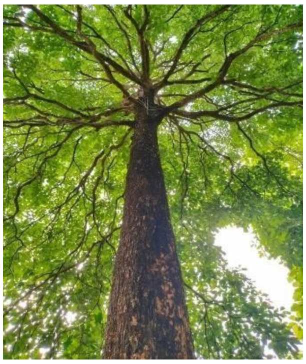
FCRI TB5

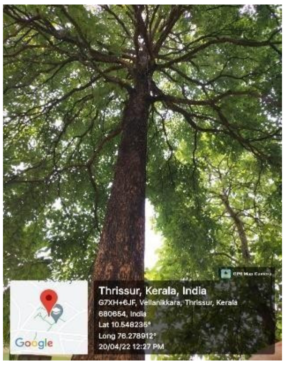

FCRI TB6

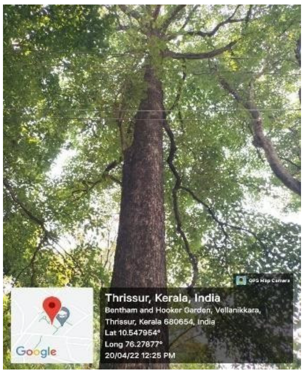

FCRI TB7

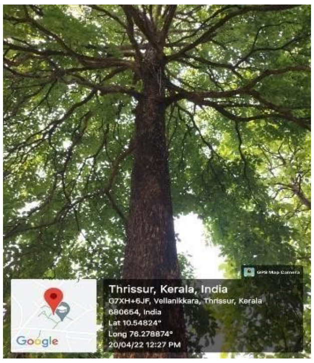

FCRI TB8

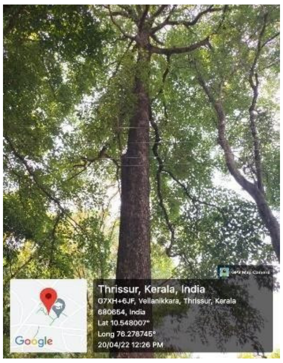

FCRI TB9

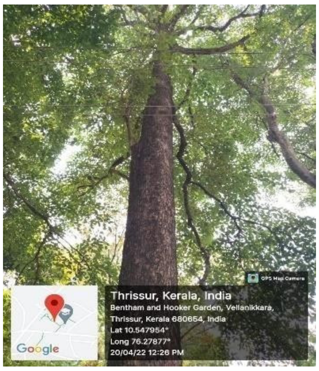

FCRI TB10

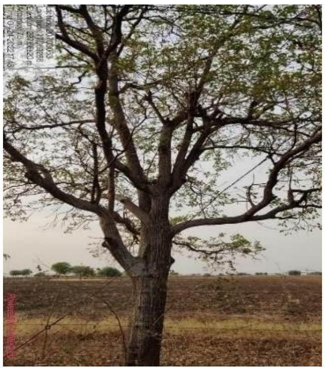
FCRI TB11

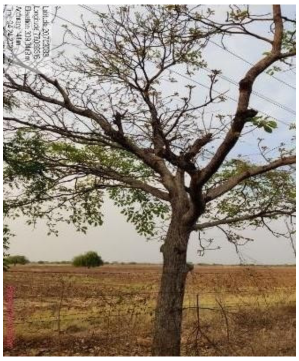
FCRI TB12

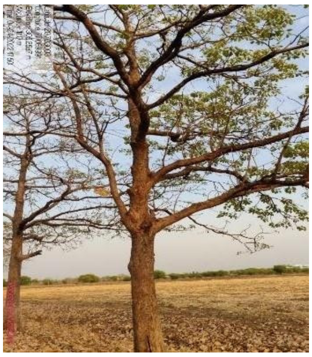

FCRI TB13

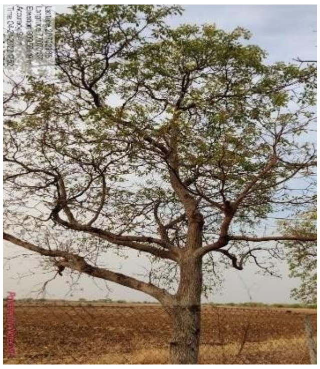

FCRI TB14

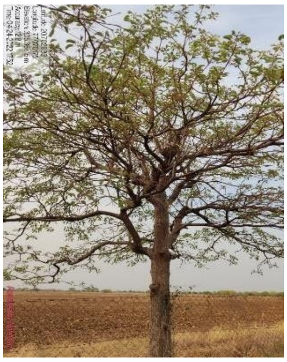

FCRI TB15

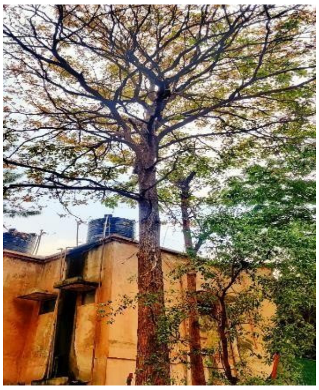

FCRI TB16

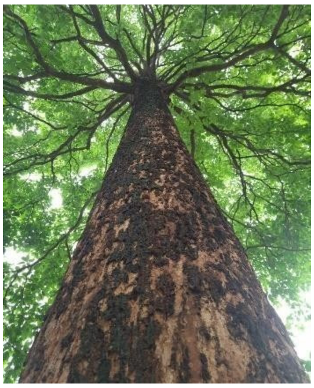

FCRI TB17

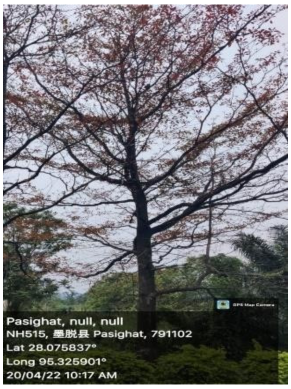

FCRI TB18

Figure S5. Selection of candidate plus

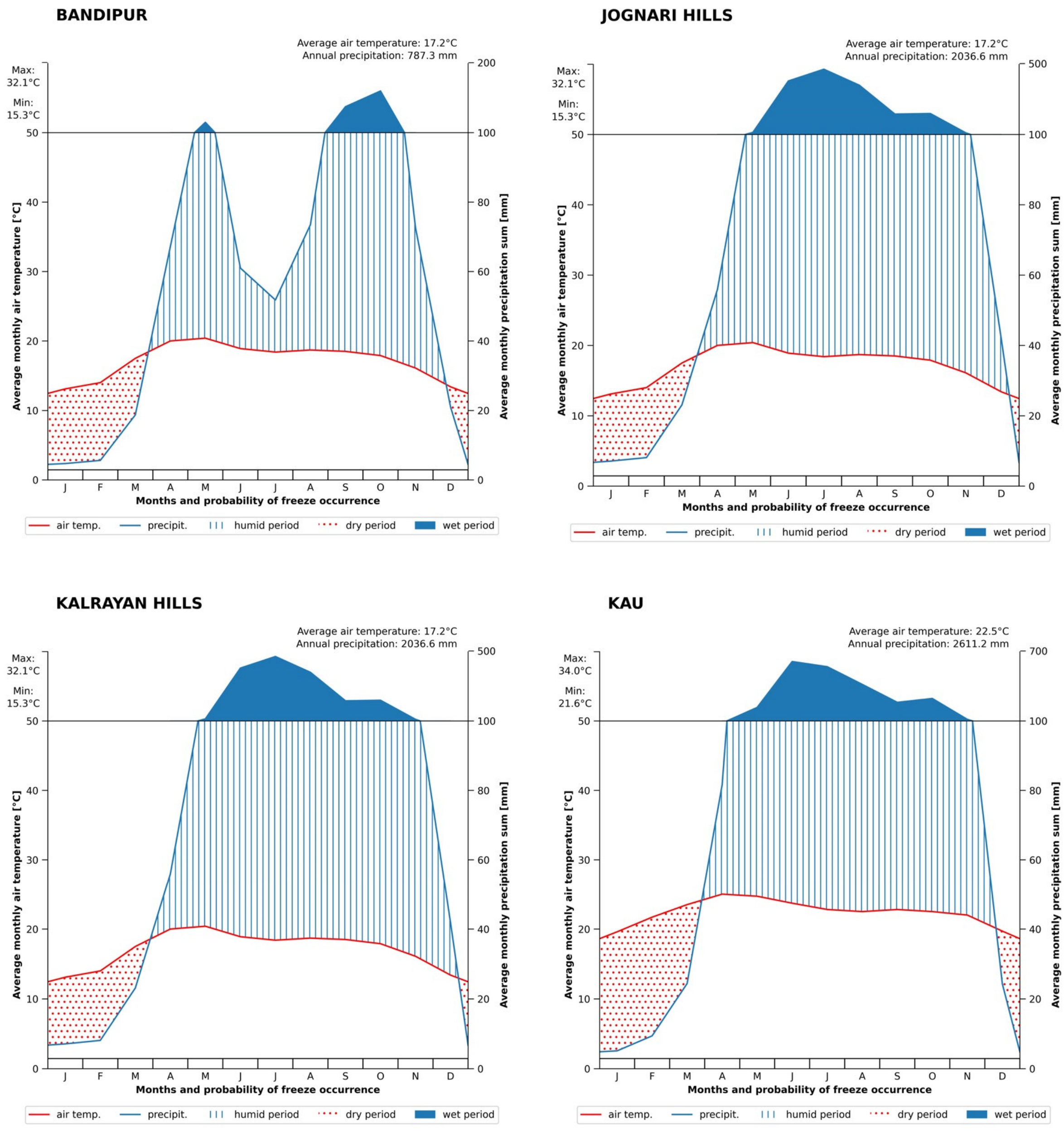

Figure S6. Walter-Leith diagram of the monthly rainfall and daily average temperature of Bandipur (Karnataka), Thrissur (Kerala), Kallakurichi (Tamil Nadu) and Jognari (Tamil Nadu).

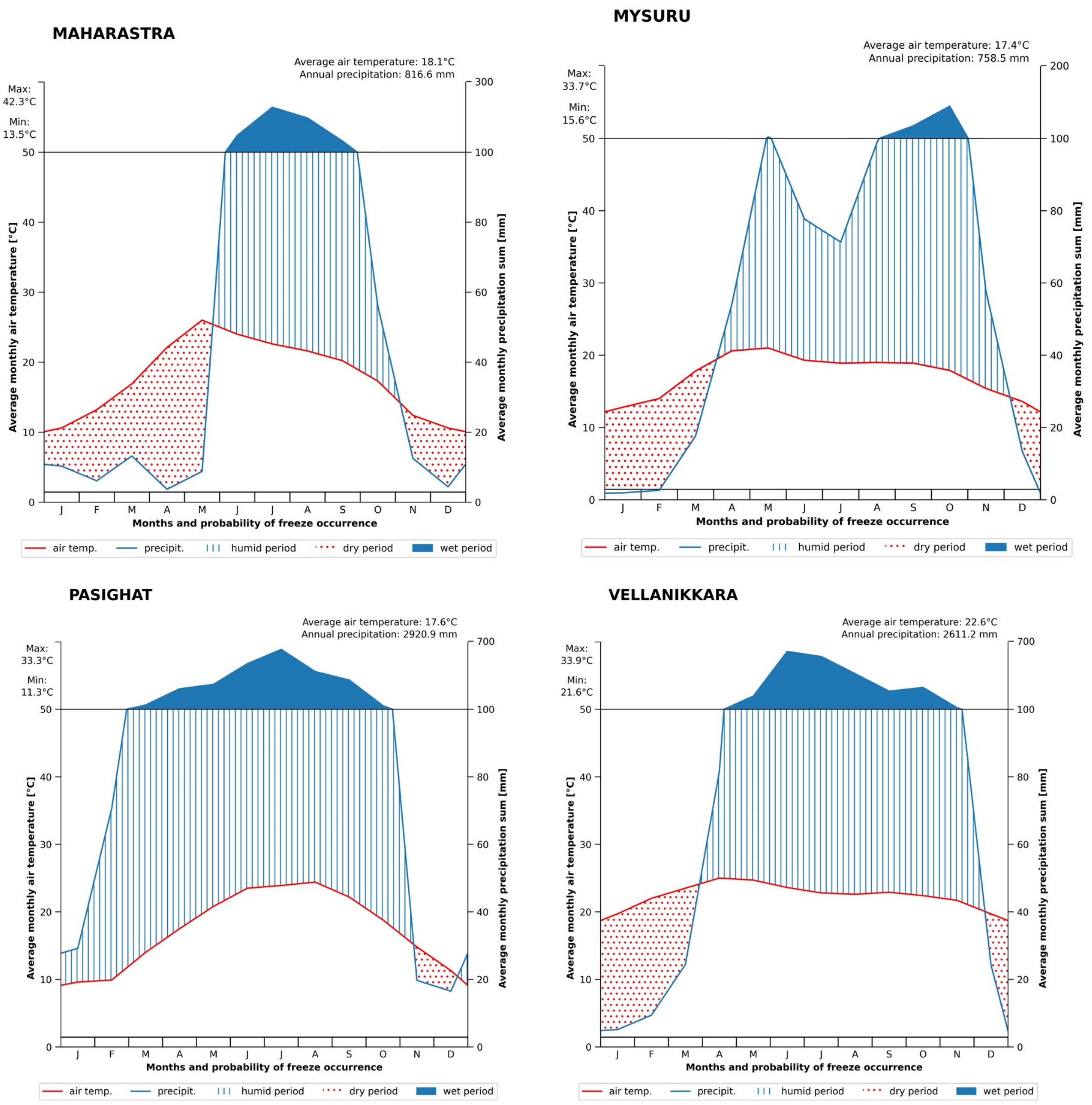

Figure S7: Walter-Leith diagram of the monthly rainfall and daily average temperature of Maharashtra, Mysuru (Karnataka), Pasighat (Arunachal Pradesh) and Vellanikkara (Kerala)
